# Mathematical models of TCR initial triggering

**DOI:** 10.3389/fimmu.2024.1411614

**Published:** 2024-07-18

**Authors:** Jiawei Shi, Weiwei Yin, Wei Chen

**Affiliations:** ^1^ Department of Cardiology, The Second Affiliated Hospital, School of Medicine, Zhejiang University, Hangzhou, China; ^2^ Key Laboratory for Biomedical Engineering of the Ministry of Education, College of Biomedical Engineering and Instrument Science, Zhejiang University, Hangzhou, China; ^3^ Zhejiang Provincial Key Laboratory of Cardio-Cerebral Vascular Detection Technology and Medicinal Effectiveness Appraisal, Zhejiang University, Hangzhou, China; ^4^ Department of Cell Biology, School of Medicine, Zhejiang University, Hangzhou, China; ^5^ Liangzhu Laboratory, Zhejiang University, Hangzhou, China

**Keywords:** T cell receptor (TCR), TCR triggering, TCR models, mathematical model, kinetic proofreading model

## Abstract

T cell receptors (TCRs) play crucial roles in regulating T cell response by rapidly and accurately recognizing foreign and non-self antigens. The process involves multiple molecules and regulatory mechanisms, forming a complex network to achieve effective antigen recognition. Mathematical modeling techniques can help unravel the intricate network of TCR signaling and identify key regulators that govern it. In this review, we introduce and briefly discuss relevant mathematical models of TCR initial triggering, with a focus on kinetic proofreading (KPR) models with different modified structures. We compare the topology structures, biological hypotheses, parameter choices, and simulation performance of each model, and summarize the advantages and limitations of them. Further studies on TCR modeling design, aiming for an optimized balance of specificity and sensitivity, are expected to contribute to the development of new therapeutic strategies.

## Introduction

T cells play pivotal roles in adaptive immunity. Their activation is initially triggered by T cell receptors (TCRs) recognizing foreign or non-self peptides presented by major histocompatibility complex (MHC) molecules on the surface of antigen presenting cells (APCs) (1, 2). The accurate antigen recognition by TCRs guarantees the immune system precisely targets and eliminates foreign pathogens or transformed cells. Previous studies have revealed that TCRs possess the capability to detect rare foreign antigens from high quantities of self antigens, endowing the immune system with ultra sensitivity to detect infected or aberrant cells (3). The antigen discrimination of TCRs is achieved usually within a time window of a few minutes, requiring the speed of recognition process to be fast (4). Furthermore, TCRs’ antigen recognition is also highly specific, safeguarding immune surveillance and preventing the development of autoimmune diseases or impaired immune responses induced by unwanted recognition of self antigens. Thus, the study of natural rules governing TCR antigen discrimination system, achieving high speed, high sensitivity, and high specificity, has long been a central issue in the field of adaptive immunity (5).

Based on accumulated experimental studies, several hypotheses have been proposed to explain the regulatory mechanism in TCR antigen discrimination. Previous studies have considered the interactions between TCR and peptide-MHC (pMHC) molecules as the major determinant in the discrimination process. Typically, the binding affinities of TCR-pMHC complexes make primary contributions to TCR specificity (6). The interactions can induce a series of modifications, such as the sequential intracellular phosphorylation of immunoreceptor tyrosine based activation motifs (ITAMs) on CD3 subunits of TCR, serving to magnify the differences of TCR signal cascade between an agonist and an antagonist (7, 8). Additionally, TCR triggering involves multiple mechanisms, such as the cooperation of coreceptors, the phosphatases regulated negative feedback, the mechanically-chemically coupled regulation of TCR and pMHC engagement, etc (9–13). Experimental evidence has demonstrated the lifetime of TCR-pMHC complex acting as the “threshold” to discriminate foreign and self peptides (14–16). Self antigens typically dissociate rapidly from TCRs (short lifetime), while foreign antigens can establish more endurable interactions (long lifetime) when engaged with TCRs. Moreover, TCRs engineered by affinity-mature strategy have been reported to induce off-target cytotoxicity, indicating the high binding affinity of TCR and pMHC may impair TCR’s specificity (17). However, some ligands with shorter lifetimes can also trigger T cell responses (18, 19). Recent studies have revealed the mechanical force regulated mechanism for TCR antigen recognition (20–24). Using the single-molecule force experiment, Liu and his colleagues found that TCR with antigenic pMHC can form a “catch-bond” with an extended bond lifetime under optimal force (20, 25). In contrast, self peptides can only form “slip-bond”. Following this “catch-bond” mechanism, Zhao et al. proposed a biophysically based strategy called “catch bond engineering to tune high sensitive TCRs” for T cell therapy with reduced cross-reactivity potentials (26). Overall, the TCR signaling system is an intricate network involving multiple molecules, reactions, and mechanisms, each alone remains insufficient to interpret the characteristics of TCR signaling comprehensively (27), but together they can explain the rapid and reliable recognition of foreign antigens by specific TCR (1, 28, 29).

To better understand the dynamic features of the TCR antigen recognition system and identify key regulators, mathematical modeling techniques have been applied. Several models have been proposed based on experimental observations, including phenotypical models such as the serial engagement model, coreceptor scanning model, conformation change model, kinetic proofreading model, and kinetic segregation model, etc. (30–32) Mathematical modeling allows for computer-aided simulations, enabling the translation of these biological models into quantitative representations. This facilitates the elucidation of TCR initiation characteristics and prediction of TCR responses to diverse stimuli (33, 34). By incorporating quantitative experimental data on protein-protein interactions, phosphorylation events, and intracellular signaling cascades, these mathematical models not only help decipher the intricate networks governing TCR signaling, but also unravel the underlying regulatory mechanisms (35–39). Moreover, these models can also be utilized to inspire novel experimental designs and interventions that modulate TCR signaling. This can lead to improved manipulation of immune responses and potentially enhance the efficacy of immunotherapies. For instance, predicting TCR responsiveness to diverse antigens can aid in screening out neoantigens, designing cancer-targeting chimeric antigen receptors (CARs) and T cell receptor engineered T cells (TCR-Ts), or recombined antigens for use in vaccines, thereby contributing to the development of novel immunotherapies applied to clinics (40–43). In the following, we will briefly summarize the relevant mathematical models with a particular focus on the kinetic proofreading models, which have been widely used to mimic TCR initial triggering.

## Kinetic proofreading model

**Figure 1:**
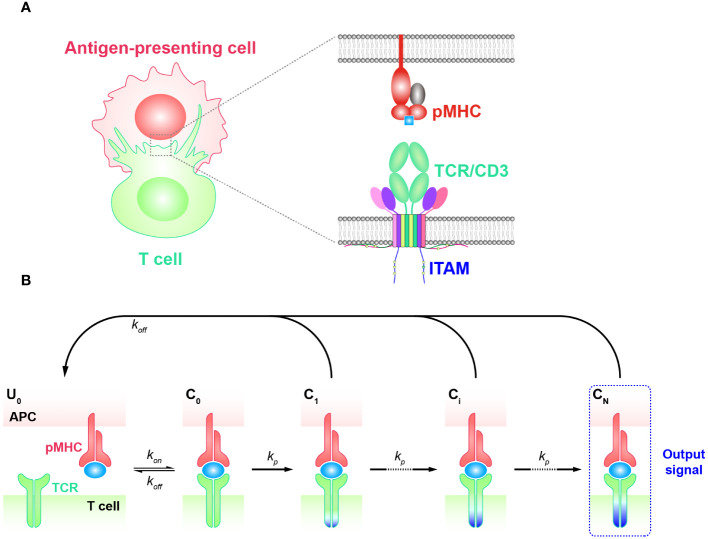
Basic kinetic proofreading model **(A)** The structural schematic diagram of TCR and pMHC in the unbound state ( $U_{0}$ ). The phosphorylation of ITAMs primarily represents the intermediate steps of the KPR model. **(B)** Schematic diagram of the basic kinetic proofreading model. The successive steps ( $C_{i}$ , *i* = 1, 2, …, N) occur after the TCR and pMHC on the antigen-presenting cell (APC) form the complex ( $C_{0}$ ) at an association rate ( $k_{on}$ ). The TCR undergoes these steps at a forward rate ( $k_{p}$ ) until the last step ( $C_{N}$ ) as the output signal. Each state of the complex ( $C_{i}$ , *i* = 0, 1, 2, …, N) can reverse to the unbound state ( $U_{0}$ ) at a dissociation rate ().

The kinetic proofreading (KPR) model describes a classic phenomenon wherein the effective signal is not immediately generated when a ligand initially engages with a receptor. Instead, there are several intermediate steps involving the phosphorylation of tyrosine residues. This process typically begins with the binding of the ligand and receptor to form a complex (44, 45) (**Figure 1A**). The KPR model was first proposed by McKeithan in 1995 to study the mechanism of TCR antigen recognition and signal transduction (46). It suggests that the pMHC initially binds to a TCR and forms the complex that then undergoes a series of intermediate steps to become an activated complex. Many of these steps involve the phosphorylation of immunoreceptor tyrosine based activation motif (ITAM) sites on intracellular CD3 subunits and require the participation of multiple molecules. This includes the recruitment of zeta-chain associated protein kinase 70 kDa (ZAP-70), the activation of tyrosine kinases such as Src family kinase (SFKs) Lck, and the participation of co-stimulators (CD27, CD28, and ICOS, etc.), coreceptors as CD8 or CD4, and phosphatases, etc. (27, 47–49). These events in concert determine whether the downstream signaling cascade is able to generate efficient signals and activate T cell responses. Consequently, a time delay exists between the initial binding and the transmission of the signal. As a result, non-specific antigens typically form short-lived complexes with TCRs, then fail to elicit downstream signals before dissociation. On the other hand, specific antigens (foreign or non-self antigens) that form long-lived complexes generate more durable signals compared to non-specific antigens. This enables the recognition and discrimination of corresponding antigens (6, 50–52).

### Basic KPR model

The basic KPR model postulates that once the TCR-pMHC complex forms, it will undergo a series of intermediate states, potentially involving modifications of the complex through intracellular tyrosine phosphorylation. Dissociation of the modified complex ( $C_{i}$ , *i* = 0, 1, 2, …, N) can lead to either reversal or directly recycled back to the unmodified state ( $U_{0}$ ), for instance, through the action of phosphatases. A productive signal is transduced only after several such modifications have taken place (**Figure 1B**).

To quantitatively analyze the basic KPR model, numerous assumptions and simplifications must be made due to the lack of knowledge about the relevant rate constants for most intermediate steps. In the hypothesis of basic KPR model, McKeithan assumed that the intermediate steps occurred in an obligatory sequential order and can go back toward the initial state (unmodified state) at a constant rate, namely the proofreading loop. Under this assumption, they investigated how the dissociation rates (off-rate, $k_{off}$ ) impacted on regulating the output signal of TCR initial triggering (53). Through the steady-state analysis, they revealed that a few fold difference in dissociation rates could lead to a thousand times difference in generated signals, demonstrating the high specificity of TCR antigen recognition. In other words, the dissociation rates of TCRs from pMHCs with self antigens need to be sufficiently high to allow for dissociation before the TCR-pMHC complex reaches the final state and generates signals. Moreover, the association rates (on-rate, $k_{on}$ ), which describes how fast TCRs can bind to pMHCs, work synergistically to affect the signal production. As a consequence, the affinity ( $K_{a}\;=\;k_{on}/k_{off}$ ), is proposed to directly quantify the potency of a pMHC triggering TCRs. According to the basic KPR model, antigens with high binding affinities (faster on-rates or smaller off-rates) would result in longer durations of TCR/pMHC interaction and generate stronger effective signals (46). It is worth noting that the affinities characterized by $k_{on}$ and $k_{off}$ can be obtained experimentally (54, 55). Three-dimensional (3D) assays are traditionally used to quantify the kinetics for TCRs and pMHCs interactions, including surface plasmon resonance (SPR), 3D fluorescence resonance energy transfer (FRET), and single-molecule fluorescent microscopy (SMFM) (56–60). As these methods usually assess the off-rates or the affinities between TCRs and pMHCs in solution, the corresponding kinetics are thus referred to as 3D kinetics. Most experimentally obtained kinetics for KPR models belong to this category. However, the binding strength measured by 3D assays often does not align with the functional output of T cells, which may be influenced by the different environmental conditions in solution compared to the *in vivo* cell-cell contact membrane environment (61). As a result, two-dimensional assays including adhesion frequency, thermal fluctuation, and 2D FRET have been developed to measure the kinetics of TCR-pMHC interactions between two contacting surfaces (referred to as 2D kinetics), which have shown better consistency with the ligand potency for T cell activation (52, 62–65). Even with the improvements in measuring kinetics, experimental studies have shown that the affinities still do not align well with the functional potency of TCRs, indicating the incompletion of the basic KPR model (66). To further understand TCR antigen recognition, researchers are tasked to consider additional regulatory factors and mechanisms within the TCR model and find approaches to optimize it. Thus, several modified models were proposed based on the KPR model.

### KPR model with fast rebinding

T cells transiently interact with APCs and decide how to respond within seconds of an encounter. The rapid turnover of T cell and APC contacts *in vivo* accelerates the search for specific but rare foreign antigens among numerous self antigens (67). Once the decision to respond is made upon the initial recognition of a specific antigen by the TCR, a stable adhesion between the T cell and APC is established, forming an immune synapse that can persist for up to 30 minutes. This immune synapse facilitates a second, sustained phase of signaling (4, 68–71). During this sustained signaling phase, the rapid engagement of many TCRs with a single pMHC is thought to increase T cell sensitivity, allowing sufficient downstream signals to accumulate within the indicated period (72, 73). T cells have been observed to respond to stimulatory pMHC in less than a minute, and a stable contact interface is not required for pMHC detection (74). Basic KPR models as aforementioned cannot predict the specificity of T cell response on such short time scales. Hence, it is difficult to determine the early T cell response based on equilibrium parameters such as the affinity or binding constant ( $K_{d}\;=\;1/K_{a}$ ), as these parameters can not precisely represent transient kinetics of TCR-pMHC interactions at the interface of T cell and APC at such short time frames. Therefore, a putative mechanism was suggested for antigen discrimination during the early phase of TCR signaling, arguing that after the complex dissociation, the TCR and pMHC molecules remain in close proximity and rapidly rebind without changing the signaling state (75, 76). This modified topology of the basic KPR model accounts for the persistence of TCR signaling when in proximity. Thus, KPR model with fast rebinding was proposed with the additional assumption that TCR can rapidly rebind to pMHC after the dissociation at much higher rates over the initial on-rate.

**Figure 2:**
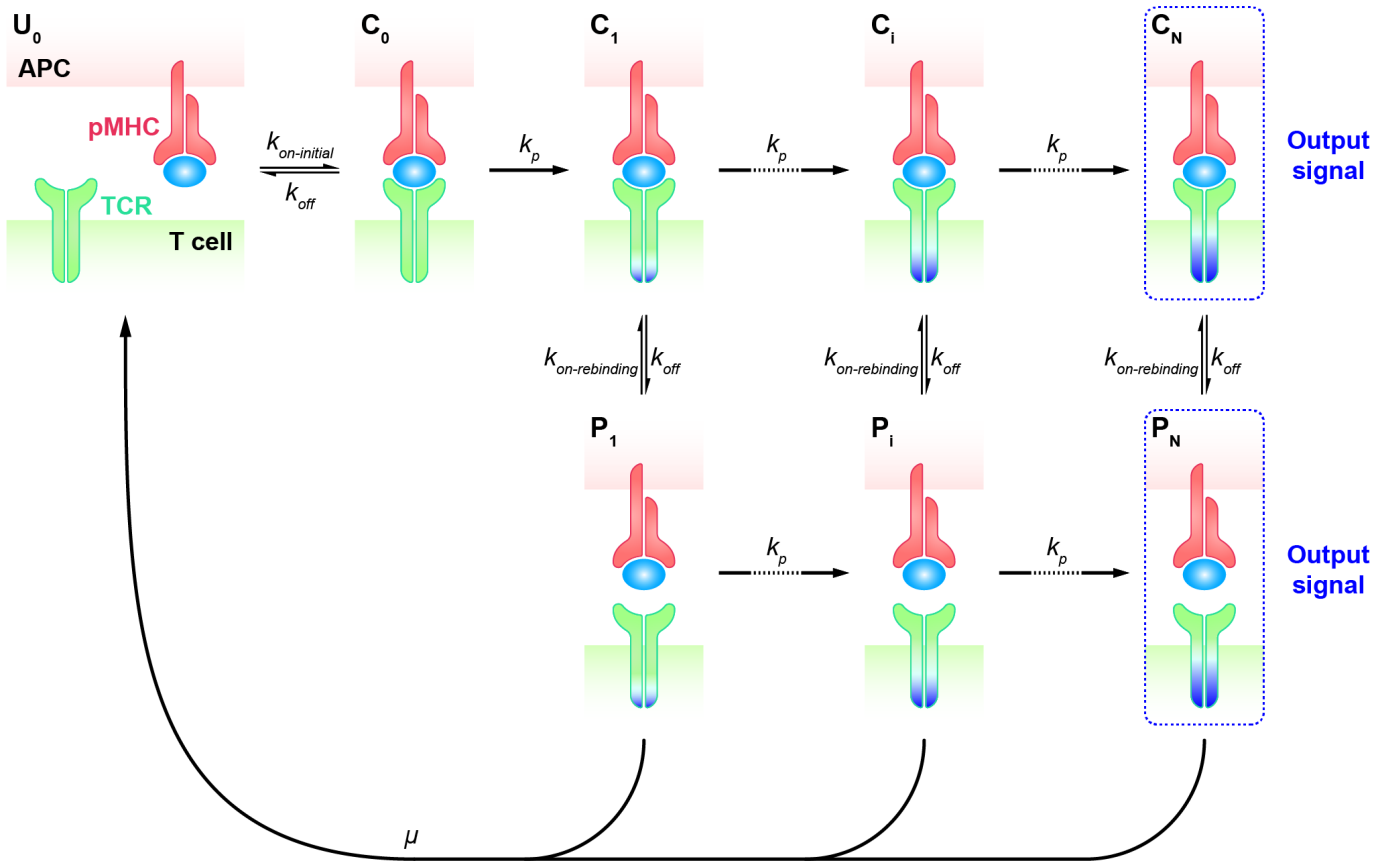
Kinetic proofreading model with fast rebinding TCR and pMHC on the APC form the complex ( $C_{0}$ ) at an initial association rate ( $k_{on-initial}$ ) and reverse to the unbound state ( $U_{0}$ ) at a dissociation rate ( $k_{off}$ ). The TCR undergoes the successive steps including intermediate complex ( $C_{i}$ , *i* = 0, 1, 2, …, N-1) and modified proximity ( $P_{i}$ , *i* = 1, 2, …, N-1) at a forward rate ( $k_{p}$ ) until the last step ( $C_{N}$ and $P_{N}$ ) as the output signal. The modified state of the complex ( $C_{i}$ , *i* = 1, 2, …, N) can dissociate into the proximal state ( $P_{i}$ , *i* = 1, 2, …, N) at a dissociation rate ( $k_{off}$ ) and rapidly re-associate at an association rate ( $k_{on-rebinding}$ ).

Specifically, the KPR model with fast rebinding suggests that the modified TCR-pMHC complex dissociates into the intermediate state in proximity ( $P_{i}$ , *i* = 1, 2, …, N). In this state, pMHC and TCR are biochemically unbound but remain physically close and can quickly reassociate through fast rebinding. Due to its unchanged biochemical state intracellularly, TCR in $P_{i}$ state can either react forward to a further modified state ( $P_{i+1}$ ) or recycle back to the unbounded and unmodified state ( $U_{0}$ ) (**Figure 2**). The transient dissociation between pMHC and TCR extracellularly does not immediately impact TCR’s intracellular state and reaction. Thus, the parameters representing the forward reaction rates for both TCR-pMHC complex ( $C_{i}$ , *i* = 1, 2, …, N) and unbounded TCR in $P_{i}$ state can be set in the same magnitude. The parameter representing the reaction rate of fast rebinding ( $k_{on-rebinding}$ ), which is very hard to be obtained experimentally, is generally thought to be relevant to the initial on-rate ( $k_{on-initial}$ ). It has been proposed and indirectly quantified to be much faster, ensuring that it is consistently large enough to enable the required model performance in TCR sensitivity and specificity (18).

To address antigen discrimination at short time scales, instead of steady-state analysis, a set of ordinary differential equations (ODEs) are utilized to describe the time-series dynamics in TCR initial triggering. The computed probability of the productive signal after a short period (e.g., t = 30 seconds) is considered as a read-out of the T cell response. By applying both the ODEs and the spatial Monte-Carlo simulations, Dushek and his colleagues investigated the capability of TCR antigen discrimination based on the values of $k_{on-initial}$ and $k_{off}$ (76). The assumed signal persistence of TCR corresponding to the “fast rebinding” process allows individual TCR to integrate the duration of multiple rebinding events. This “sum-of-binding” mechanism not only captures rapid and reliable T cell responses to specific pMHC, but also leads to enhanced sensitivity to the initial on-rate ( $k_{on-initial}$ ), whereas the basic KPR model for TCR recognition has a trade-off between specificity and sensitivity. Incorporating the “fast rebinding” mechanism into basic KPR model amplifies the discrimination of antigens, and optimizes the specificity and sensitivity of TCRs without introducing cross-reactivities. For instance, an order of magnitude change in the initial on-rate can enhance the probability of productive signal by several orders of magnitude. This model with heightened sensitivity promotes T cells to discriminate a wider spectrum of antigens than would be predicted by a traditional serial engagement/KPR model.

### KPR model with coreceptor

**Figure 3:**
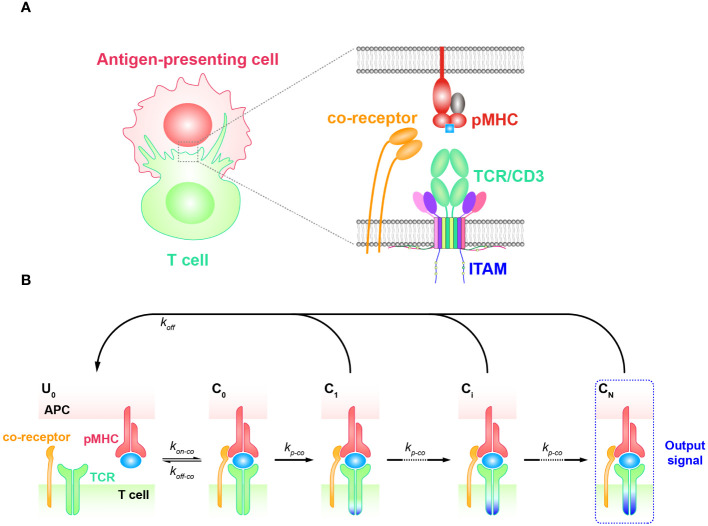
Kinetic proofreading model with coreceptors **(A)** The structural schematic diagram of TCR and pMHC in the unbound state ( $U_{0}$ ). The phosphorylation of ITAMs primarily represents the intermediate steps of the KPR model. **(B)** Schematic diagram of the basic kinetic proofreading model. The successive steps ( $C_{i}$ , *i* = 1, 2, …, N) occur after the TCR and pMHC on the APC form the complex ( $C_{0}$ ) at an association rate ( $k_{on-co}$ ). The TCR undergoes these steps at a forward rate ( $k_{p-co}$ ) until the last step ( $C_{N}$ ) as the output signal. Each state of the complex ( $C_{i}$ , *i* = 0, 1, 2, …, N) can reverse to the unbound state ( $U_{0}$ ) at a dissociation rate ( $k_{off-co}$ ).

Coreceptors, such as CD4 and CD8, are also important players facilitating TCR antigen discrimination and T cell activation (77–81). When TCRs bind to antigenic pMHCs, these coreceptors are involved in the recognition process both extracellularly and intracellularly (82, 83) (**Figure 3A**). Previous research has shown that the extracellular domains of CD8 can bind to different regions of MHC molecules, facilitating TCRs to effectively scan the surface of APCs for searching cognate antigens (78, 84, 85). Besides, CD8 can also stabilize the binding of TCR and pMHC for cognate antigens, increasing the probability of T cell activation in response to weak antigenic stimuli (16, 86). In addition, they can promote TCR to dissociate from non-cognate antigens, preventing T cell activation in response to irrelevant stimuli. Thereafter, TCR coreceptor scanning model is proposed to explain this mechanism, suggesting that TCRs bind to the pMHCs through a dynamic equilibrium of binding and unbinding events (87). The binding process is facilitated by the coreceptors, which can bind to the MHC molecules in different conformations and modulate the binding kinetics of the TCR to cognate antigens. The TCR coreceptor scanning mechanism assists T cells to recognize and respond to a wide range of antigens presented by pMHCs more efficiently (87).

The intracellular domains of coreceptors can recruit activated Src family kinases, specifically Lck, and thus facilitate the phosphorylation of tyrosine residues in the CD3 intracellular domain (88, 89). This phosphorylation is crucial for transmitting downstream signaling in T cell activation. Thus, KPR model with coreceptor was proposed with the additional assumption that coreceptors assist in amplifying the TCR discrimination power of diverse ligands. In order to incorporate the effect of coreceptors, some studies chose to alter the topology of basic KPR model. For instance, Dushek and his colleagues have postulated a modified KPR model that orchestrates both the participation of coreceptors and fast rebinding process, and this model suggests that coreceptors can assist TCR to be more sensitive to weak pMHCs. However, the altered topology of the basic KPR model may introduce more parameters that cannot be obtained by experiments, thus the artificial manual parameters may puzzle the regulatory mechanism of coreceptors in TCR signal transmission (75). On the other hand, instead of modifying the model structure itself, the effect of coreceptors can otherwise be reflected in changing the parameter values accordingly. For instance, the addition of coreceptors can change the values of the binding kinetics between TCR-coreceptor and pMHCs ( $k_{on-co}$ and $k_{off-co}$ ) and the forward rate ( $k_{p-co}$ ) of the KPR model, respectively (**Figure 3B**) (90–92). However, there also exist limitations for this method. While it is possible to experimentally measure the binding kinetics for trimolecules (TCR, coreceptor, and pMHC), it remains difficult to clearly determine the reaction order as well as their kinetics between TCR and pMHC or between coreceptor and MHC (48).

### KPR model with negative/positive feedback

Although the KPR model amplifies the kinetic differences of TCRs in response to altered peptides, allowing for their discrimination, it typically requires a sufficient number of forward steps and a noticeable dependence on the dissociation rate of the TCR-pMHC complex. Furthermore, the open loop characteristics of basic KPR model are not suitable for explaining the observed antagonism phenomenon, wherein the presence of antagonists can suppress the TCR response to agonists. Therefore, modified KPR models have been proposed to incorporate negative/positive feedback involving multiple mechanisms (93–97). In the KPR model with negative/positive feedback, it’s assumed that two competing feedback pathways (the positive feedback pathway ERK activation and the negative pathway SHP-1 activation) exist wherein TCR-pMHC interactions trigger the MAPK cascade as a high-gain digital amplifier inducing a swift SHP-1-mediated negative feedback and a slower digital ERK-1-dependent positive feedback shaping the digital threshold of T cell activation.

**Figure 4:**
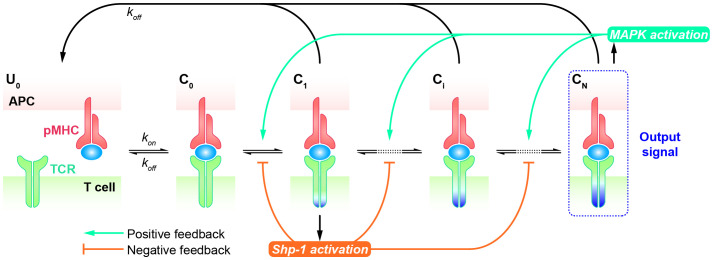
Kinetic proofreading model with positive/negative feedbacks The successive steps ( $C_{i}$ , *i* = 1, 2, …, N) occur after the TCR and pMHC on the APC form the complex ( $C_{0}$ ) at an association rate ( $k_{on}$ ). The TCR undergoes these steps until the last step ( $C_{N}$ ) as the output signal. The detailed network of positive and negative feedback pathways can be referred in Ref.98. Each state of the complex ( $C_{i}$ , *i* = 0, 1, 2, …, N) can reverse to the unbound state ( $U_{0}$ ) at a dissociation rate ( $k_{off}$ ).

The Altan-Bonnet and Germain groups modified the basic KPR model by adding digital positive feedback based on ERK activity and analog negative feedback involving Src homology 2 domain phosphatase-1 (SHP-1) (**Figure 4**) (98–100). They used an elaborate model with hundreds of variables and equations to define a sharp ligand discrimination threshold while preserving a rapid and sensitive response. This model also showed that the threshold is highly sensitive to delicate alterations in SHP-1 expression levels. Another molecular model was proposed to elucidate experimental observations for extremely low concentrations of agonists by adding feedback regulation on the relevant KPR kinases (e.g., Lck) (101). Although this model explained the disparate observations, particularly for the co-existence of agonist and antagonist, it is also quite complex with up to 50 parameters, and has to be solved stochastically. Based on these previous studies, Paul Francois et al. proposed a phenotypic model for early T cell activation that relies on the basic KPR model with only the SHP-1 negative feedback (32). Compared with the aforementioned studies, this model can simultaneously explain how TCR discriminates the rare foreign peptides rapidly from a great many self peptides, and fits a larger range of experimental data with minimal variables and parameters. This model can largely be solved analytically and does not require any cooperativity between self and foreign peptides at low concentrations of agonists. Using this simplified model, Paul Francois and his colleagues explained the counterintuitive response induced by weak agonists with high concentrations and tied it to the activity of the phosphatase SHP-1. Besides, they also characterized antagonistic effects as a trade-off for antagonism between antagonist lifetime and concentration. Using the same model structure, Guillaume Gaud et al. tested the role of ITAM multiplicity in TCR signaling and accurately predicted a non-monotonicity of antagonism depending on the affinity of the antagonist ligand (27).

## Discussion

The antigen recognition of TCR is a complicated process involving many factors and has been intensively studied in the past few decades. However, the underlying mechanism governing this process is not completely clear up to now. Most studies have agreed that high speed, high sensitivity, and high specificity are the most essential characteristics for TCR triggering. “High speed” means that the recognition signal can be generated very quickly, within seconds (9, 102, 103), after the initial engagement of TCR and pMHC. This characteristic is ultrasensitive to the magnitude of reaction rates in simulation. It can be easily achieved by adjusting the parameters, such as the forward reaction rate of TCR-pMHC complex ( $k_{p}$ ), or the rebinding reaction rate ( $k_{on-rebinding}$ ) of disassociated TCR and pMHC molecules, etc. As for the characteristics of “high sensitivity” and “high specificity”, they are assumed to be essential to ensure the accurate recognition triggered by non-self or foreign antigens while avoiding unnecessary side effects induced by non-specific recognition of cognate antigens. However, till now, the mathematical models of TCR triggering always result in a trade-off of these two properties. In other words, the increase of TCR sensitivity often comes at the cost of reducing its specificity, and vice versa. This is also the general limitation of KPR models. Thus, different modified KPR models are proposed to achieve either high sensitivity or high specificity, or both in a compromised manner.

Another general limitation of KPR models lies in their assumption of an equal reaction rate for intermediate steps. It is still unknown whether all the intermediate steps share the same forward and backward reaction rates. So, this type of parameters in KPR models cannot be experimentally measured and heavily depends on the hypothesis in each study. For instance, to simplify the simulation, the forward rate constants for all intermediate steps are often assumed to have the same value (75, 76). Another type of parameters, such as the on-rate or off-rate of TCR and pMHC binding, can be obtained explicitly by experimental measurement directly or indirectly. For example, as aforementioned, the off-rate is the reciprocal of the bond lifetime of TCR-pMHC interacting, and the on-rate can be calculated by the experimentally measured affinity ( $K_{a}$ ) or binding constant ( $K_{d}$ ). Different studies applied different experimental setups to characterize these parameters, which may lead to magnitude difference of the parameter values (21, 61, 75, 76).

Besides, “time scale” and “dynamic nature of cellular environment” are also two important factors that differ in different KPR models. The signal caused by TCR antigen recognition not only occurs rapidly, but also can be maintained and transduced downstream for a longer period of time. For instance, an increase in intracellular calcium signal can be detected on the time scale of several minutes (102, 103). The clustering of TCRs happens upon the engagement of ligands in a few minutes, helping to sustain the TCR downstream signaling (104, 105). Thus, different models also vary greatly in simulation time scale, depending on their own study purposes. As a result, the one focusing on the time scale of “second” usually cannot explain the signals or biological events that occur at the minute or even longer time scale, which represents another type of model limitation. Moreover, the dynamic nature of the cellular environment, such as the movement of the cytoskeleton, the clustering of TCRs, etc., are not considered by all KPR models. The fast rebinding KPR model partially considered this by introducing the fast rebinding step, which allows the dissociated pMHC and TCR molecules in proximity can rebind very quickly without changing the intracellular state of TCRs.

All of these existing mathematical models have been developed to explain the TCR antigen recognition behavior, aiming to find the canonical rule that can simultaneously possess the properties of high speed, high sensitivity, and high specificity. However, up to now, none of these models can mimic the three properties simultaneously as realistic TCR behavior. Instead, each model would rather focus on distinct but crucial procedures of TCR antigen recognition, such as the participation of coreceptors, the feedback from downstream signal (e.g., ERK activation pathway), etc. Thus, selecting the proper mathematical model in one’s study preferably depends on the research priority of the TCR discrimination process, correspondingly resulting in the strength and limitations of each model. One of the major issue of current TCR modeling is the lack of the generic criteria for model comparison. The properties, such as speed, sensitivity, and specificity, can not be compared across different models quantitatively with a unified standard. As in different models, or even for the same model in different studies, different initial conditions, experimental data, as well as model assumptions, were used to characterize the same property. For instance, both experimental 2D and 3D assays were used to estimate the off-rate of TCR and pMHC interaction, leading to the magnitude difference of this parameter (52, 61). Some studies take into account the concentration of pMHCs and TCRs, while others focus on the reaction at the single-molecule level, resulting the different calculations for specificity and sensitivity (75, 76, 98). As for prediction accuracy, different labs or studies often used different sets of experimental data for evaluations. These experimental functions of TCRs were either measured by different functional assays (e.g., IL-2 releasing or calcium influx, etc.) or measured for different TCR systems that seldom have consistent observations (102, 103, 106, 107). Therefore, the evaluation of each model was conducted only on their own or selected data, and the comparisons between models are hard to implement due to the lack of standardized and harmonized TCR functional data.

Taking advantage of the various KPR models mentioned above to investigate TCR initial triggering, we have gained a deeper understanding of how TCRs recognize foreign or non-self antigens and initiate corresponding T cell responses in a fast, sensitive, and specific manner (108). Nevertheless, there remain unsolved questions that require further exploration. For instance, recent studies have uncovered the importance of mechanical force in regulating the TCR and pMHC interaction (22, 23). Under force, an agonist can form the “catch-bond” with TCR where increasing the force strengthens the binding of TCR and pMHC until excessive force overpowers the bond, turning it into a slip bond. While an antagonist only forms the “slip-bond” with TCR where increasing the force shortens the lifetime of TCR-pMHC. Therefore, different TCR-pMHC pairs have different patterns of force-dependent bond lifetime (represented by the reciprocal of off-rate, $1/k_{off}$ ). As T cells can exert approximately 10-20 pN force at the molecular level (109), it is possible to accurately estimate the off-rate of TCR-pMHC binding under the force in physiological conditions. Although currently there are no TCR models adopting these force-dependent kinetics, Fan, et al. have proposed an NKG2D model by using the force-dependent disassociation rate. Their simulation results have demonstrated that the force-dependent affinity had better discrimination power for NKG2D as compared to in solution kinetics or *in situ* affinity measured without force, suggesting the possibility and effectiveness of integrating force-dependent kinetics into the KPR models (110). Moreover, exploring how T cells integrate multiple cues to generate different signals in different contexts is also essential for understanding their role in maintaining homeostasis of the immune system. Hitherto, the relationship between single-cell-level and population-level T cell responses is still not fully understood. Most established mathematical models have used the 3D kinetic parameters obtained from 3D assays rather than the more physiologically significant 2D kinetic parameters that are measured by 2D assays, such as thermal fluctuation or 2D FRET, etc. As a consequence, in order to get a more reliable simulation, these models need to be updated and iterated with 2D kinetic parameters. Furthermore, other regulatory players (e.g., co-stimulatory and co-inhibitory molecules) have not yet been added to the current mathematical model due to unclarified regulatory mechanisms, although they have been reported to be involved in regulating T cell responses.

Using experimental observations and computer-aided simulations, mathematical models have the potential to elucidate the key players and molecular reactions that govern TCR signaling initiation. This, in turn, will facilitate the identification of prospective therapeutic targets for immune-related diseases and disorders. Further study regarding the TCR model design with optimized specificity and sensitivity will undoubtedly shed light on new therapeutic strategies for immune-related disorders.

## Author contributions

JS: Writing – original draft, Writing – review & editing. WC: Funding acquisition, Resources, Supervision, Writing – review & editing. WY: Funding acquisition, Resources, Supervision, Writing – review & editing.
